# Neuroplasticity of cognitive control networks following cognitive training for chronic traumatic brain injury^☆^

**DOI:** 10.1016/j.nicl.2018.01.030

**Published:** 2018-01-31

**Authors:** Kihwan Han, Sandra B. Chapman, Daniel C. Krawczyk

**Affiliations:** a Center for BrainHealth®, School of Behavioral and Brain Sciences, The University of Texas at Dallas, Dallas, TX, USA; b Department of Psychiatry, University of Texas Southwestern Medical Center, Dallas, TX, USA

**Keywords:** Traumatic brain injury, Rehabilitation, Cognitive function, Resting-state functional connectivity, Neuroplasticity, Cognitive control

## Abstract

Cognitive control is the ability to coordinate thoughts and actions to achieve goals. Cognitive control impairments are one of the most persistent and devastating sequalae of traumatic brain injuries (TBI). There have been efforts to improve cognitive control in individuals with post-acute TBI. Several studies have reported changes in neuropsychological measures suggesting the efficacy of cognitive training in improving cognitive control. Yet, the neural substrates of improved cognitive control after training remains poorly understood. In the current study, we identified neural plasticity induced by cognitive control training for TBI using resting-state functional connectivity (rsFC). Fifty-six individuals with chronic mild TBI (9 years post-injury on average) were randomized into either a strategy-based cognitive training group (N = 26) or a knowledge-based training group (active control condition; N = 30) for 8 weeks. We acquired a total of 109 resting-state functional magnetic resonance imaging from 45 individuals before training, immediately post-training, and 3 months post-training. Relative to the controls, the strategy-based cognitive training group showed monotonic increases in connectivity in two cognitive control networks (i.e., cingulo-opercular and fronto-parietal networks) across time points in multiple brain regions (*p*_voxel_ < 0.001, *p*_cluster_ < 0.05). Analyses of brain-behavior relationships revealed that fronto-parietal network connectivity over three time points within the strategy-based cognitive training group was positively associated with the trail making scores (*p*_voxel_ < 0.001, *p*_cluster_ < 0.05). These findings suggest that training-induced neuroplasticity continues through chronic phases of TBI and that rsFC can serve as a neuroimaging biomarker of evaluating the efficacy of cognitive training for TBI.

## Highlights

• We examined training-induced changes in cognitive control networks in chronic TBI.
• Cognitive training increased CON and FPN connectivity over time.
• FPN connectivity of the training group was correlated with the trail-making scores.
• Training-induced neuroplasticity continues through chronic phases of TBI.
• RsFC may serve as a biomarker for the efficacy of cognitive training for TBI.

## 1 Introduction

A traumatic brain injury (TBI) occurs when external force is applied to the head leading to disruptions of brain structure and function (Faul et al., 2010). Though an insult to the brain occurs instantaneously, a TBI incident can be the beginning of a chronic disease process rather than an isolated event or final outcome across all levels of initial injury severity: moderate or severe TBI (Corrigan et al., 2014; Masel and DeWitt, 2010; Whitnall et al., 2006) and mild-to-severe TBI (Masel and DeWitt, 2010; Whitnall et al., 2006). For example, TBI can be a risk factor for cognitive impairments (Arciniegas et al., 2002; Rabinowitz and Levin, 2014), psychiatric disorders (Hesdorffer et al., 2009), reduced social functioning (Temkin et al., 2009), and neurodegenerative diseases such as chronic traumatic encephalopathy (McKee et al., 2013). A substantial number of individuals with TBI sustain TBI-related disabilities. For example, 57% of individuals 16 years or older with moderate or severe TBI were moderately or severely disabled, and 39% had a worse global outcome at 5 years post-injury compared to their outcome level at 1 or 2 years post-injury (Corrigan et al., 2014). Currently, as many as 5.3 million people in the U.S. are facing challenges of TBI-related disability (Frieden et al., 2015). The actual number of individuals continuing to suffer from chronic TBI (>6 months post-injury time) effects may be greater than the estimates given the lack of public awareness of TBI in the past and the limited sensitivity of conventional neuropsychological measures (Katz and Alexander, 1994). Additionally, conventional clinical imaging (e.g., CT scanning) may be insensitive to identifying brain abnormalities especially in individuals with mild TBI (Tellier et al., 2009). Substantial numbers of individuals with sustained TBI necessitates further rehabilitation research in chronic TBI (Katz and Alexander, 1994).

Resting-state functional connectivity (rsFC) is a technique measuring the temporal coherence of blood oxygenation level dependent (BOLD) signal from anatomically separated brain regions acquired at rest. Since its inception (Biswal et al., 1995), rsFC in resting-state functional magnetic imaging (rsfMRI) has provided new insights about brain networks that can better explain the underlying mechanisms of human behavior or function (van den Heuvel and Hulshoff Pol, 2010). RsFC studies in clinical populations are increasingly popular because they do not require that subjects perform a specific task. RsFC is well-positioned to identify both the patterns of injury and the associations between injury and behavioral impairments in TBI (Sharp et al., 2014). This is especially important as diffuse axonal injury (DAI) is one of the primary injury mechanisms of TBI (Smith et al., 2003). DAI induces multi-focal injuries to axons which provide the structural basis of spatially distributed brain networks. Thus, DAI leads to a breakdown of brain network connectivity. In the context of rehabilitation, rsFC is also a promising technique to measure neuroplasticity within the *injured* brain, as rsFC has been successfully utilized to provide evidence for experience-induced neuroplasticity of the adult human brain in vivo (Guerra-Carrillo et al., 2014; Kelly and Castellanos, 2014). For example, in healthy subjects, previous studies reported changes in rsFC after motor training (Lewis et al., 2009; Taubert et al., 2011), cognitive training (Jolles et al., 2013; Mackey et al., 2013; Takeuchi et al., 2013), and physical activity in older adults (Voss et al., 2010). In clinical populations, changes in rsFC after cognitive rehabilitation for cognitive symptoms associated with multiple sclerosis has been reported (de Giglio et al., 2016; Keshavan et al., 2017). This technique is well-suited to investigating neuroplasticity induced by rehabilitation for TBI.

In a previous study, we reported the efficacy of strategy-based cognitive training for chronic TBI, utilizing neuropsychological measures (Vas et al., 2016). This training is an integrative program to improve cognitive control by exerting more efficient thinking strategies for selective attention and abstract reasoning (see the Materials and methods section for the details of training protocols). Cognitive control (also called executive function) is the ability to coordinate thoughts and actions to achieve goals while adjusting these goals according to changing environments (Nomura et al., 2010). Cognitive control is critical to successfully perform daily life tasks (Botvinick et al., 2001; Diamond, 2013). Thus, impairment in cognitive control is one of the most persistent and devastating sequalae of TBI (Cicerone et al., 2000; Rabinowitz and Levin, 2014), and empirical studies demonstrating the efficacy of cognitive rehabilitation for improving cognitive control of individuals with post-acute TBI are valuable in the literature on TBI rehabilitation (Cicerone et al., 2006; McDonald et al., 2002). In the current study, we describe rehabilitation-induced changes in brain connectivity.

Cognitive control has been extensively investigated in the field of cognitive neuroscience (Power and Petersen, 2013). Of note, Dosenbach and colleagues (Dosenbach et al., 2006) identified a set of regions that are active across multiple cognitive control tasks. A follow-up study (Dosenbach et al., 2007) revealed two distinct resting-state networks related to cognitive control: the cingulo-opercular network and fronto-parietal network. The cingulo-opercular network consists of bilateral anterior insula/frontal opercula (aI/fO), bilateral anterior prefrontal cortices (aPFC), dorsal anterior cingulate cortex (dACC), and thalamus, and it is thought to support stable maintenance of task mode and strategy during cognitive processes (Dosenbach et al., 2007, Dosenbach et al., 2008). The fronto-parietal network comprises of bilateral dorsolateral prefrontal cortices (dlPFC), bilateral dorsal frontal cortices (dFC), bilateral inferior parietal lobules (IPL), bilateral intraparietal sulci (IPS), middle cingulate cortex (mCC), and bilateral precunei (PCUN), supporting active, adaptive online control during cognitive control processes (Dosenbach et al., 2007, Dosenbach et al., 2008). The cingulo-opercular network and fronto-parietal network are also referred to as the salience network and central executive network, respectively (Seeley et al., 2007). The salience and central executive networks are often referred to in the context of interactions among these networks and the default mode network (Menon and Uddin, 2010). However, in this report, we will refer to them as the cingulo-opercular and fronto-parietal networks, as we conducted current study in the context of cognitive control. TBI-induced disruptions to the cingulo-opercular network in mild-to-severe TBI (Bonnelle et al., 2012; Jilka et al., 2014; Stevens et al., 2012) and fronto-parietal network in mild TBI (Mayer et al., 2011; Stevens et al., 2012) have been previously reported. Specifically, TBI decreases the white matter integrity of the cingulo-opercular network (Bonnelle et al., 2012) and functional connectivity between the cingulo-opercular and default networks during a cognitive control task (Jilka et al., 2014). Additionally, individuals with mild TBI showed increases and decreases in rsFC with the cingulo-opercular (Stevens et al., 2012) and fronto-parietal networks (Mayer et al., 2011; Stevens et al., 2012) across brain regions, relative to healthy individuals.

We utilized rsfMRI to identify the effects of a strategy-based cognitive training for chronic TBI on the cognitive control networks (i.e., cingulo-opercular and fronto-parietal networks) compared to a knowledge-based comparison condition. We focused on the cingulo-opercular and fronto-parietal networks as our training protocols were aimed at improving cognitive control processes (See the Materials and methods section for the details of training protocols). We randomized individuals with chronic mild TBI into two eight-week training groups (strategy- versus knowledge-based), and we acquired their MRI scans over three time points (prior to training, after training, and at three-months follow-up after training completed). We then investigated the spatial and temporal patterns of training-induced changes in cingulo-opercular and fronto-parietal networks connectivity of these individuals. We hypothesized that strategy-based cognitive training would induce changes in the cingulo-opercular and fronto-parietal networks connectivity relative to the knowledge-based training program. This prediction is based on findings from previous rsfMRI studies demonstrating neuroplasticity in healthy adults and other clinical populations (de Giglio et al., 2016; Jolles et al., 2013; Keshavan et al., 2017; Lewis et al., 2009; Mackey et al., 2013; Takeuchi et al., 2013; Taubert et al., 2011; Voss et al., 2010) and the efficacy of strategy-based cognitive training for chronic TBI (Vas et al., 2016).

## 2 Materials and methods

### 2.1 Participants

We selected a subset of 83 individuals with chronic TBI from a larger study (Krawczyk et al., 2013). Participant selection criteria included a diagnosis of mild TBI and no visible focal lesions or extreme degeneration of the white matter on structural MRI scans when MRI scans were available. We excluded 26 participants whose estimated initial injury severity was moderate or severe. Further, we excluded one participant with abnormally low premorbid intelligent quotient. After the selection procedure, we analyzed 56 individuals at the chronic stage of mild TBI who ranged from lower moderate disability to lower good recovery (age 20–65; >6 months post-injury; 5–7 on the Extended Glasgow Outcome Scale (Wilson et al., 1998)).

The rsfMRI data from 45 participants were included for rsFC analyses, as ten participants did not have MRI scans and one participant did not pass the selection criteria for structural MRI scans (i.e., data quality and no visible abnormalities). Participants were recruited from the Dallas–Ft. Worth community. Demographic data and TBI screening information was obtained during a phone screening interview before inclusion in the study. The primary causes of TBIs in this group were blasts, blunt force trauma, falls, athletic impacts, vehicle accidents or combinations thereof. Initial injury severity was retrospectively estimated utilizing the Ohio State University TBI identification (OSU TBI-ID) method (Corrigan and Bogner, 2007). The rationale for utilizing the OSU TBI-ID and the details of the OSU TBI-ID is described in our previous study (Han et al., 2017). Both civilian and veteran participants were included (see Table 1 for demographics). No participants had a history of any significant, clinically-diagnosed neurological or psychiatric comorbidities. We also confirmed that all participants were free from visible focal brain lesions or extreme degeneration of the white matter on structural MRI scans. This confirmation should minimize the potential effects of such macrostructural injuries on preprocessing for rsFC analyses. All participants provided written informed consent, and this study was conducted in compliance with the Declaration of Helsinki. The study was approved by the Institutional Review Boards of the University of Texas at Dallas and University of Texas Southwestern Medical Center.

**Table 1:** Participant demographics by group after quality assurance procedures.

Demographics	SMART	BHW	*p*-Values
Number of subjects	26	30	–
Gender (male, female)	16, 10	19, 11	>0.1
Civilians, Veterans	17, 9	18, 12	>0.1
Age (years)^a^	40.5 ± 14.0	42.8 ± 12.4	>0.1
Education (years)^a^	15.5 ± 2.2	15.9 ± 2.2	>0.1
Current IQ	108.4 ± 11.6	111.3 ± 10.0	>0.1
Premorbid IQ	108.5 ± 9.6	111.6 ± 8.2	>0.1
BDI-II^b^	23.3 ± 9.3	18.5 ± 12.6	>0.1
PCL-S^b^	46.4 ± 16.5	46.5 ± 19.0	>0.1
Post-injury time (years)^a^	8.1 ± 9.0	10.4 ± 9.6	>0.1
Primary cause of injury (blast, blunt force trauma, fall, athletic impacts, vehicle accidents, combined)	5, 2, 2, 8, 7, 2	5, 6, 5, 5, 7, 2	>0.1

*Note*: SMART, Strategic Memory Advanced Reasoning Training; BHW, Brain Health Workshop; IQ, Intelligent Quotient; BDI-II, Beck Depression Inventory-II; PCL-S, Post-traumatic Stress Disorder Check List Stressor-specific.

a Mean and standard deviation values were reported.

b None of the participants were clinically diagnosed with depression or PTSD.

### 2.2 Training protocols

All participants were randomly assigned to one of the two training groups: (1) a strategy-based reasoning training called Strategic Memory Advanced Reasoning Training (SMART) group (N = 26) or (2) the knowledge-based training called Brain Health Workshop (BHW) group (N = 30). The BHW group served as an active control condition. Both training programs comprised of 12 sessions (1.5 h per session) for 8 weeks with quizzes, homework assignments, and projects conducted in small group settings, comprised of 4–5 participants per group. Briefly, the SMART group focused on selective attention, abstract reasoning, and other thinking strategies (Vas et al., 2011). The BHW group focused on information about brain structure and function and the effects of sleep and exercise on the brain performance (Binder et al., 2008). More specifically, the SMART participants were trained to (1) manage information by blocking distractions and irrelevant information, along with avoiding multitasking, (2) increase the ability to understand overall ideas and actionable messages from information, and (3) examine information from divergent perspectives. This set of strategies was aimed at improving cognitive control over incoming information. The SMART strategies were introduced using slides by one of two trained clinicians. Each of the strategies were sequentially introduced while these were reinforced throughout the training sessions. Example materials that were used in order to practice the learned strategies include newspaper articles and audio-video clips. The BHW participants learned about brain anatomy, brain function, the effects of a TBI on cognitive function, neuroplasticity, and the impact of diet, physical exercise, sleep, and social activities on brain health through slides led by one of the clinicians. The participants were also encouraged to discuss the application of learned information to their daily lives. The number of participants was equivalent in each training session to control for the effects of social activities on training outcomes. Both training programs were conducted at The University of Texas at Dallas Center for BrainHealth®. Refer to our previous study (Vas et al., 2016) for more detailed descriptions of the SMART and BHW programs.

### 2.3 Neuropsychological assessments

We selected a subset of tests from the full testing battery (Krawczyk et al., 2013). The selected tests are relevant to performance of executive functions and showed training-induced improvement (Vas et al., 2016). These tests include card-sorting and trail-making from the Delis-Kaplan Executive Function System (D-KEFS) for problem solving and processing speed (Delis et al., 2001). From the card-sorting test, we selected correct sorts and description scores during free sorting and description scores during sort recognition. From the trail-making test, we selected scores on number-letter switching versus motor speed. As the three selected scores from the card sorting test were highly correlated, we obtained composite scores by averaging the sub-scores.

To determine the efficacy of training compared to healthy individuals, we obtained scaled scores for the card-sorting and trail-making tests from the D-KEFS manual (Delis et al., 2001) and assessed these scaled scores by the types of training at each time point. Unlike raw scores, the scaled scores allowed us to compare the neuropsychological performance of the participants relative to normative samples. Specifically, at each assessed time point, we identified participants with reduced neuropsychological performance relative to healthy individuals by counting the percentage and number of the participants who performed below the average range of the normative samples (less than scaled scores of 7; lower than one standard deviation from the mean).

In addition, we acquired full scale intelligent quotient-2 (FSIQ-2) from the Wechsler Abbreviated Scale of Intelligence for estimated current IQ (Wechsler, 1999) and FSIQ from the Wechsler Test of Adult Reading for estimated premorbid IQ (Wechsler, 2001). While the participants did not have clinically significant psychiatric conditions, individuals with TBI often have some degree of non-clinically significant psychiatric symptoms (Ashman et al., 2004; Hibbard et al., 1998; van Reekum et al., 1996). Thus, for more thorough characterization of the participants, we quantified *subclinical-but-residual* depressive and post-traumatic stress disorder (PTSD) symptoms severity of the participants by measuring the Beck Depression Inventory-II (BDI-II; Beck et al., 1996) and PTSD Check List Stressor-specific (PCL-S; Weathers et al., 1993). Note that, unlike carding-sorting and trail-making tests, BDI-II and PCL were not primary measures of interest to identify differential effects of training.

### 2.4 MRI data acquisition

We acquired MRI scans of the participants at three time points: prior to training (TP_1_), after training (TP_2_) and 3 months later (TP_3_). The participants underwent MRI scans in a Philips Achieva 3T scanner (Philips Medical Systems, Netherlands) in the Advanced Imaging Research Center at the University of Texas Southwestern Medical Center. In each imaging session, T_1_-weighted sagittal Magnetization Prepared Rapid Acquisition Gradient Echo (MPRAGE) images were acquired using a standard 32-channel head coil (Repetition Time (TR)/Echo Time (TE) = 8.1/3.7 m; Flip Angle (FA) = 12°; Field of View (FOV) = 25.6 × 25.6 cm; matrix = 256 × 256; 160 slices, 1.0 mm thick). In this imaging session, either one or two 416-s runs of rsfMRI scans were also acquired using the same head coil with T_2_^⁎^-weighted image sequence (TR/TE = 2000/30 ms; FA = 80°; FOV = 22.0 × 22.0 cm; matrix = 64 × 64; 37 slices, 4.0 mm thick). The total number of rsfcMRI runs differed across the participants because, at the early stage of our study, we observed that the quality assurance (QA) procedures with only one rsfMRI run yielded high rates of participant exclusion. Thus, we additionally acquired two rsfMRI runs for the remainder of the data collection. Refer to the rsfMRI data analysis section for our strategy to account for differences in total number of rsfMRI scans across the participants. During rsfMRI acquisition, the participants were asked to remain still with their eyes closed.

### 2.5 RsfMRI preprocessing

We used AFNI (Cox, 1996) to preprocess rsfMRI data. Each subject's structural images were first skull-stripped and registered to the Montreal Neurological Institute (MNI) space (Evans et al., 1993). For each rsfMRI run, we discarded the initial four time points. Then we applied despiking, slice timing correction, motion correction, coregistration to the structural images in the MNI space using a single affine transform with spatial resampling (4 mm isotropic), normalization to whole brain mode of 1000, and simultaneous band-pass filtering (0.009 < f < 0.08 Hz) and linear regression. In the linear regression, we 2nd order detrended the rsfMRI time-series and regressed out six parameters for the rigid body head motion acquired from the motion correction, the signal averaged over the lateral ventricles and deep cerebral white matter, respectively, the first temporal derivatives of all these parameters (i.e., motion, ventricles, and white matter), and the squared motion parameters. Further, we incorporated regressors for bandpass filtering in this nuisance regression to prevent from reintroducing high frequency oscillations into the rsfMRI signals (Hallquist et al., 2013). To account for temporal autocorrelation of rsfMRI signals, we also applied the pre-whitening procedure during the nuisance regression (Bright et al., 2017). To reduce motion-related confounds (Scheinost et al., 2014), we iteratively smoothed the remaining rsfMRI signals until the smoothness of image reached at 12 mm full-with-at-half-maximum (FWHM) using AFNI's 3dBlutToFWHM with the spatial autocorrelation function (ACF) option. We chose this smoothness level because 12 mm FWHM with ACF option was close to the minimum smoothness of rsfMRI signals across scans when conventional a 6 mm FWHM Gaussian kernel smoothing was applied. This uniform smoothing procedure was shown to control the effects of subject motion during scanning without removing high-motion timeframes (Scheinost et al., 2014). See the Control analyses section to confirm whether the uniform smoothing procedure controlled for motion-related effects. If two rsfMRI runs were acquired, we selected one of the two preprocessed rsfMRI runs which had less amount of head motion, measured by frame-wise displacement (FD; Power et al., 2012).

### 2.6 Seed-based connectivity analysis

**Fig. 1:**
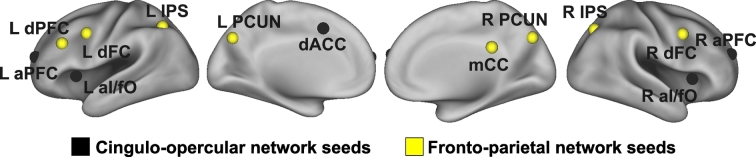
Seed locations. Black and yellow circles represent seeds for the cingulo-opercular network and fronto-parietal network, respectively. aI/fO, anterior insula/frontal operculum; aPFC, anterior prefrontal cortex; dACC, dorsal anterior cingulate cortex; dFC, dorsal frontal cortex; dlPFC, dorsolateral prefrontal cortex; IPS; intraparietal sulcus; mCC, middle cingulate cortex; PCUN, precuneus; L, left; R, right.

We identified cingulo-opercular and fronto-parietal networks connectivity by seeding a 5 mm radius sphere at each of the five cingulo-opercular network regions predominantly associated with set-maintenance control signal and the eight fronto-parietal network regions predominantly associated with start cue-related control signal (Dosenbach et al., 2007, Dosenbach et al., 2008). The five cingulo-opercular network seeds included the bilateral aI/fO (L: −35, 14, 5; R: 36, 16, 4), bilateral aPFC (L: −28, 51, 15; R: 27, 50, 23), and dACC (−1, 10, 46). The eight seeds of the fronto-parietal network include the bilateral dFC (L: −41, 3, 36; R: 41, 3, 36), left dlPFC (−43, 22, 34), bilateral IPS (L: −31, −59, 42; R: 30, −61, 39), mCC (0, −29, 30), and bilateral PCUN (L: −9, −72, 37; R: 10, −69, 39). These seed locations were obtained from a previous study (Dosenbach et al., 2007). See Fig. 1 for locations of the seeds within the brain. For each seed, Pearson correlation maps were then Fisher's *Z*-transformed to improve the normality of correlations, followed by scaling to *z*-scores (i.e., normal distributions with zero mean and unit variance) for presentation purpose. Note that the scaling factor was same across all scans due to the same number for frames, which mathematically did not change the results when only the Fisher's Z-transformation was utilized.

To identify patterns of training-related changes in cingulo-opercular and fronto-parietal networks connectivity, we performed the linear mixed-effects model (LME; Bernal-Rusiel et al., 2013) analysis at each of the voxels using the LME MATLAB toolbox. We used the LME MATLAB toolbox (as opposed to AFNI's 3dLME) because the LME MATLAB toolbox enabled us to run LME analyses on the MRI data and neuropsychological assessment scores (3dLME does not have this capability). For this analysis we used a piece-wise linear model with a break-point at the post-training time point, a randomly varying intercept, and psychiatric symptom covariates for both within- and between-subject components. We obtained psychiatric symptom severity by averaging Z-scores for BDI and PCL-S as these measure were highly correlated (*r* = 0.69; *p* < 10^−20^) and the inclusion of both BDI and PCL-S covariates could yield the multicollinearity problem in the LME model. Although the participants did not have clinically significant depressive or PTSD-related symptoms, we included psychiatric symptom covariates as both training group showed statistically significant reductions in BDI-II and PCL-S scores (Table S1; see the Control analyses section for the effects of psychiatric symptom covariates). Mathematically, connectivity of subject *i* at time point *j*, *y*_*ij*_, can be written as:

$$y_{\mathrm{ij}}\left(t_{\mathrm{ij}}\right)=\beta_{1}+\beta_{2}\cdot t_{\mathrm{ij}}+\beta_{3}\cdot \left(t_{\mathrm{ij}}-\bar{t}\right)\cdot H\left(t_{\mathrm{ij}}-\bar{t}\right)+\beta_{4}\cdot S_{i}+\beta_{5}\cdot S_{i}\cdot t_{\mathrm{ij}}+\beta_{6}\cdot S_{i}\cdot \left(t_{\mathrm{ij}}-\bar{t}\right)\cdot H\left(t_{\mathrm{ij}}-\bar{t}\right)+\beta_{7}\cdot {\bar{P}}_{i}+\beta_{8}\cdot \left(P_{\mathrm{ij}}-{\bar{P}}_{i}\right)+b_{i}+e_{\mathrm{ij}}$$

where *t*_*ij*_ is the time of measurement for subject *i* at time point *j*, $\bar{t}$ is an average time of measurement at TP_2_, *S*_*i*_ is an indicator function for the SMART group for subject *i*, *b*_*i*_ is a subject-specific intercept (connectivity of subject *i* at TP_1_), ${\bar{P}}_{i}$ is the average psychiatric symptom score for subject *i*, *P*_*ij*_ is psychiatric symptom score for subject *i* at time point *j*, *e*_*ij*_ is measurement error for subject *i* at time point *j*, and *H*(·) is the Heaviside step function.

We performed subsequent statistical inferences for the within- and between-group contrasts of connectivity at monotonic (H_0_: (*y*(TP_2_) − *y*(TP_1_)) + (*y*(TP_3_) − *y*(TP_2_)) = *y*(TP_3_) − *y*(TP_1_) = 0) and non-monotonic (H_0_: (*y*(TP_2_) − *y*(TP_1_)) + (*y*(TP_2_) − *y*(TP_3_)) = 0) changes over the three time points. Refer to our previous study (Han et al., 2017) for a graphical description of monotonic and non-monotonic changes. We identified statistically significant training-induced temporal changes in cingulo-opercular and fronto-parietal networks connectivity from the between-group contrast over all time points at *p*_voxel_ < 0.001. A correction for multiple comparisons was applied across voxels by cluster size using AFNI's 3dClustSim with the spatial ACF option (Cox et al., 2017) at *p*_cluster_ < 0.05 (minimum 12 voxels; 768 mm^3^) with bi-sided and faces or edges nearest neighbor clustering parameters. Cluster-size was estimated using the average ACF parameters across rsFC scans (Cox et al., 2017). The average effective FWHMs across scans obtained by the spatial ACF method (AFNI's 3dFWHMx) were 11.82 mm. Because of criticism of cluster-based thresholding methods (Eklund et al., 2016), we used the most recent version of AFNI to estimate spatial ACF using a mixture model of Gaussian and mono-exponential functions, and we selected cluster-forming level (i.e., *p*_voxel_ = 0.001) demonstrated to be sufficient for controlling false positive rates (Cox et al., 2017). Local peaks were identified when local minima and maxima of statistics of between-group contrasts for changes over three time points were at least 30 mm apart. To determine which group(s) led to such statistically significant between-group differences, we then used the results of the within-group contrasts to assess changes over all three time points. If the corrected version of the within-group contrast results did not clearly reveal which group(s) led to the observed between-group differences, we performed a further assessment using an uncorrected version (*p*_voxel_ < 0.001) of the within-group contrast results.

Further, we identified whether training-induced changes in connectivity occurred within the regions where seed-based connectivity had already been established at the chronic stage of TBI, or in other regions where cognitive control networks might have been disrupted after TBI. Specifically, for each seed, we obtained a map of the surface overlap with baseline seed-based connectivity, indicating within which regions the between-group contrast for temporal changes in connectivity fell within or outside the baseline seed-based connectivity. We obtained the baseline connectivity using a contrast for baseline connectivity over both groups (i.e., H_0_: *y*(TP_1_) for SMART = 0 and *y*(TP_1_) for BHW = 0) in the above LME model.

Lastly, we assessed the patterns of training-induced changes in connectivity from the perspective of large-scale networks, often described in the resting-state functional connectivity literature. First, we obtained surface-wise union maps of group contrasts for temporal changes in seed-based connectivity within the cingulo-opercular and fronto-parietal networks, respectively. We then obtained the overlapping regions with changes in the two cognitive control networks over the cerebral cortex. Subsequently, we qualitatively identified network affiliations of the overlapping regions according to the Yeo atlas of large-scale resting-state networks (Yeo et al., 2011). We further quantified the network affiliations of the regions with changes in the two cognitive control networks by counting the number of voxels showing statistically significant changes in each seed-based connectivity according to resting-state networks. Lastly, we aggregated these voxel counts across the seeds.

### 2.7 Assessment of brain—behavior relationships

To identify whether cingulo-opercular and fronto-parietal networks connectivity were associated with neuropsychological test performance over three time points, we selected neuropsychological tests of interest, which showed prominent group differences in performance over time (see the Results section). Then we additionally included within-subject and between-subject covariates of these selected test scores in the LME model. Note that we adjusted for age, years of education, and psychiatric symptom severity scores before we included the within- and between-subject covariates in the revised LME model to prevent potential effects of these measures on neuropsychological test scores. Statistically significant associations (1) between changes in cingulo-opercular and fronto-parietal networks connectivity and improvement in neuropsychological test scores (i.e., within-subject covariate) and (2) between the average connectivity over time and the average neuropsychological test scores (i.e., between-subject covariate) were respectively identified at *p*_voxel_ < 0.001, *p*_cluster_ < 0.05 (minimum 14 voxels; 896 mm^3^) with one-sided and faces or edges nearest neighbor clustering parameters. Note that, in this analysis, we performed one-sided hypothesis test (i.e., positive association) as the SMART group showed both improvement in neuropsychological scores and increases in connectivity over time.

### 2.8 Quality assurance

We visually inspected structural MRI scans to ensure that subjects had no significant brain atrophy. In rsfMRI preprocessing, the quality of the preprocessed data was visually inspected at each step. After preprocessing, we excluded rsfMRI scans with the average FD over time >0.5 mm (Power et al., 2012). We also ensured that there were no MRI scans or neuropsychological measures that were acquired too late (i.e., outside the two-standard-deviation band from the mean) for all time points. Lastly, we excluded MRI scans from the LME analysis when corresponding BDI scores were not available. See Table 2 for the number of MRI scans after the QA procedure.

**Table 2:** The number and timing of neuropsychological assessments and MRI scans per time point by group.

Data type	Time point	SMART	BHW	Weeks from baseline
Neuropsychological assessments	TP_1_	26	30	–
	TP_2_	25	25	8.8 ± 0.8
	TP_3_	19	24	18.0 ± 1.5
Resting-state fMRI scans^a^	TP_1_	20	21	–
	TP_2_	20	19	8.7 ± 0.7
	TP_3_	14	15	20.6 ± 1.4

*Note*: TP_1_, Prior to training; TP_2_, After training; TP_3_, 3 months later.

a Only MRI scans that passed the quality assurance procedures were reported. 21 and 24 participants for SMART and BHW, respectively.

### 2.9 Statistical analyses

All statistical analyses were conducted in MATLAB R2013a. First, we performed the Shapiro-Wilk test at *α* = 0.05 to assess the normality of distributions of each group's demographics (age, years of education, post-injury time, current IQs, and premorbid IQs). Age, years of education, and post-injury time did not pass the Shapiro-Wilk normality test. Thus, the Mann-Whitney *U* test was used to compare these demographics between the groups. We performed *t*-tests to compare current and premorbid IQs between the groups. The Fisher's exact test was used to compare the gender distributions and proportion of civilians and veterans between the groups. The likelihood ratio chi-square test was used to compare the distribution of primary cause of injury between the groups. Similar to the longitudinal analysis of rsFC, we used the LME MATLAB toolbox (Bernal-Rusiel et al., 2013) to perform the LME analysis on the other neuropsychological measures using a piece-wise linear model with a break-point at TP_2_, and a randomly varying intercept. We included years of education, estimated current IQ, and psychiatric symptom severity covariates for age-adjusted scores of the card sorting test and trail making test in these analyses. The age, years of education, and estimated current IQ covariates were not included for BDI-II and PCL-S, as we did not expect the effects of age, years of education, and estimated current IQ on BDI-II and PCL-S scores. We confirmed that there were no statistically significant effects of age and years of education, and estimated current IQ on these measures.

### 2.10 Control analyses

#### 2.10.1 Motion analysis

To identify whether there were systematic differences in subjects head motion during rsfMRI scans, we performed LME analyses on the average FD of each scan. We also performed the LME analysis on connectivity with additional covariates for FD (van Dijk et al., 2012) to confirm the uniform smoothing procedure effectively controlled for head motion during rsfMRI scans.

#### 2.10.2 Assessment of the effects of injury characteristics on connectivity

We assessed the effects of residual depressive symptoms severity on our findings, we performed the LME analysis without covariates for residual psychiatric symptom severity and obtained between-group contrasts for changes in connectivity over time. To identify potential effects of diverse post-injury time on our findings, we included a covariate for post-injury time in the LME analysis and obtained between-group contrast for changes in connectivity over time.

#### 2.10.3 Assessment of the subsets of the participants

To identify whether there were potential biases in subject attrition present in our findings, we performed the LME analysis on the participants who underwent MRI scans at all three time points. To confirm that the patterns of improvement in neuropsychological performance were retained for the participants who underwent MRI scans, we also performed the LME analysis on neuropsychological measures of the subset of the participants.

### 2.11 Visualization

The thresholded volumetric statistical results for cingulo-opercular and fronto-parietal networks connectivity and covariate analyses were surface-projected onto the cortical surface of the Conte69 human surface-based atlas (van Essen et al., 2012) using a multi-fiducial mapping that avoids the biases of choosing a cortical surface from a single-individual as an atlas target, implemented in Caret Software (van Essen et al., 2001).

## 3 Results

### 3.1 Demographics

All participants were at a long-term chronic phase of TBI (approximately 9 years post-injury time on average). There were no statistically significant group differences in demographics at *α* = 0.05 (Table 1).

### 3.2 Neuropsychological measures

**Fig. 2:**
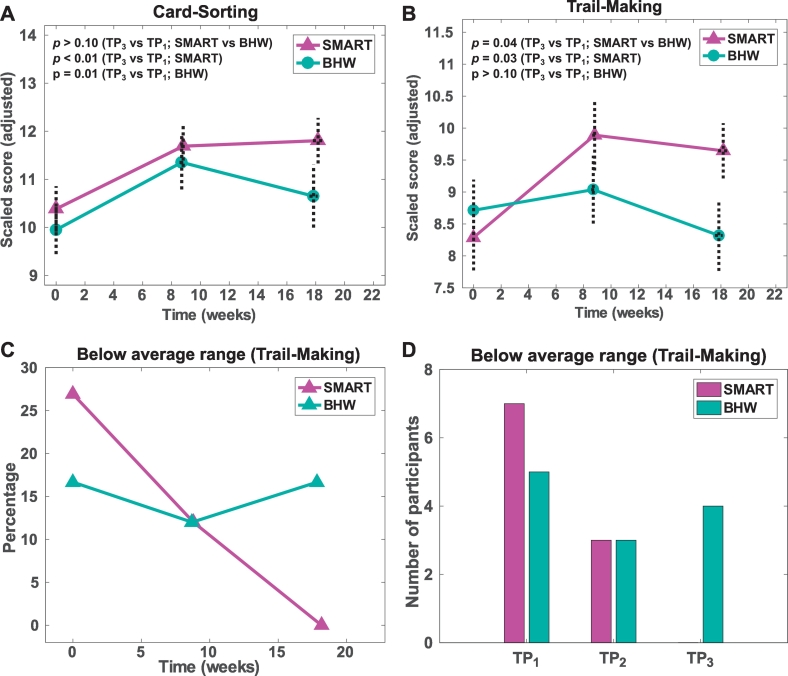
Neuropsychological assessment results. TP_1_, Prior to training; TP_2_, After training; TP_3_, 3 months later; SMART, Strategic Memory Advanced Reasoning Training; BHW, Brain Health Workshop.

The average times of assessments at TP_2_ and TP_3_ were 9 and 18 weeks from the baseline, respectively (Table 2). Interactions with current IQ were statistically significant (*p* < 0.01) for the card-sorting scores and marginally significant (*p* = 0.08) for the trail-making scores. The SMART group showed improvements in both card-sorting and trail-making scores, whereas the BHW group showed improvement only in the card-sorting scores (Table S2). Such within-group changes yielded a statistically significant (*p* = 0.04) group difference in training-induced changes over time only in trail making scores (Fig. 2; Table 3). There were no statistically significant group differences in BDI-II and PCL-S scores over time though both groups showed statistically significant reductions in these scores over time (Table S1). The percentage of the participants who underwent SMART and performed poorly relative to a normative sample was decreased from 27% at TP_1_ to 0% at TP_3_, indicating that all participants for SMART at TP_3_ performed the neuropsychological tests at a level comparable to healthy individuals (Fig. 2C–D). In contrast, the BHW group retained the participants with reduced performance across the time points.

**Table 3:** Neuropsychological assessment results.

Neuropsychological measure	SMART (N = 26)			BHW (N = 30)			*p*-Values
	TP_1_	TP_2_	TP_3_	TP_1_	TP_2_	TP_3_	(M, NM)^a^
Card-sorting (SS)	9.8 ± 2.4	11.5 ± 2.2	11.8 ± 2.1	9.9 ± 2.8	11.7 ± 2.8	11.2 ± 3.2	>0.1, >0.1
Trail-making (SS)	8.0 ± 2.6	9.8 ± 2.8	9.7 ± 1.9	8.7 ± 2.6	9.2 ± 2.7	8.5 ± 2.7	0.04^⁎^, >0.1

*Note*: SS, scaled scores; M, monotonic; MN, non-monotonic. See Table 1, Table 2 for other abbreviations.

a * represents *p* < 0.05.

### 3.3 RsfMRI analysis results

#### 3.3.1 Individual seed-based connectivity

**Fig. 3:**
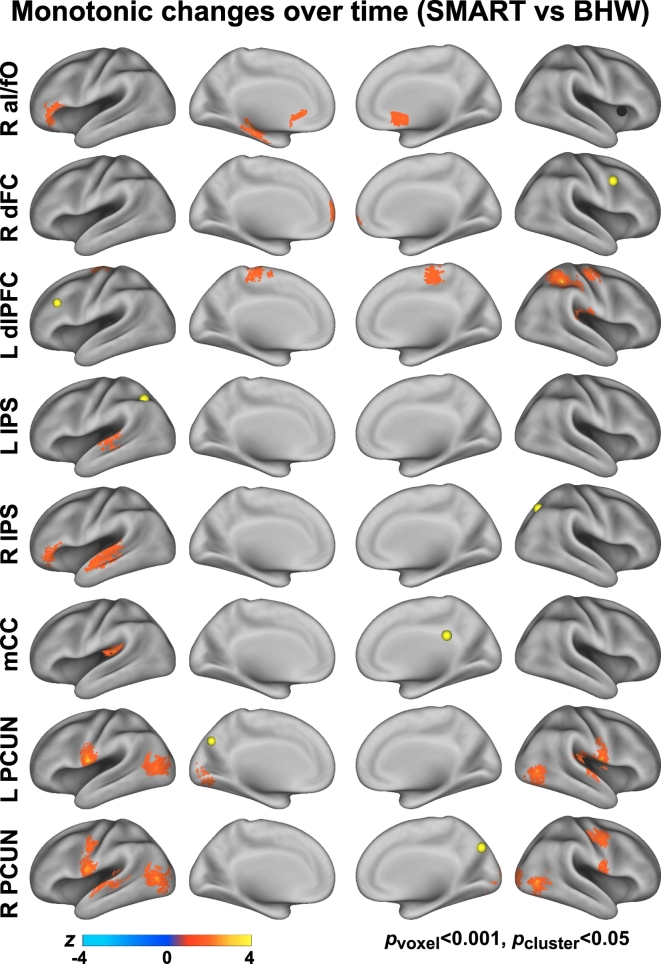
Between-group contrast maps for changes in connectivity over time. See Fig. 1, Fig. 2 for abbreviations.

The average timing of rsfMRI scan acquisition at TP_2_ and TP_3_ were 9 and 21 weeks from the baseline, respectively (Table 2). The number of rsfMRI scans within each group per time point by civilians versus veterans and primary cause of injury are summarized in Tables S3, S4. Relative to the BHW group, the SMART group showed statistically significant (*p*_voxel_ < 0.001, *p*_cluster_ < 0.05) monotonic increases in cingulo-opercular network connectivity with R aI/fO seed, and fronto-parietal network connectivity with R dFC, L dlPFC, L IPS, R IPS, mCC, L PCUN, and R PCUN seeds, respectively (Fig. 3; Table S5).

**Fig. 4:**
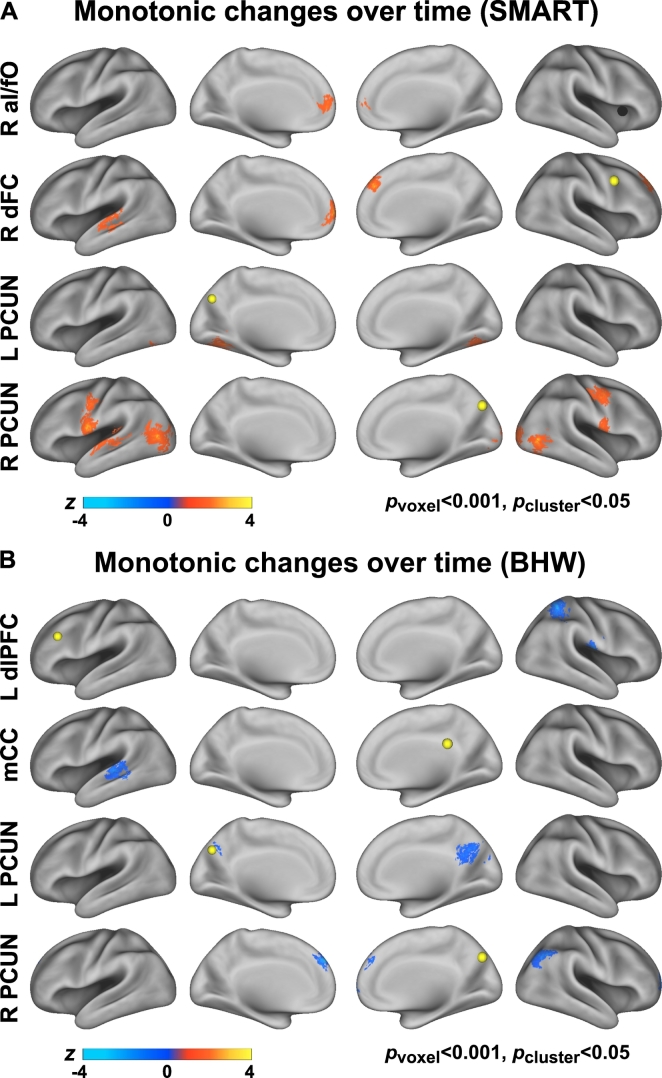
Within-group contrast maps for changes in connectivity over time.

**Fig. 5:**
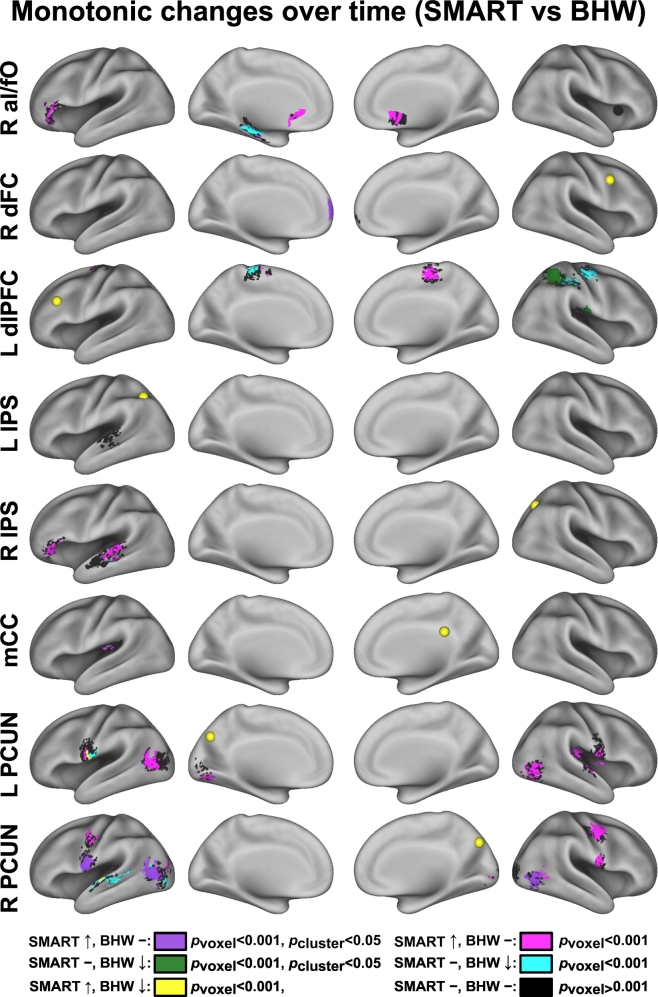
Colormaps for between-group contrast for temporal changes in connectivity according to the patterns of within-group changes.

The directionality of change should be noted, as either the SMART or BHW group can drive between-group differences. Although the within-group differences in temporal changes in cingulo-opercular and fronto-parietal networks connectivity at *p*_voxel_ < 0.001 and *p*_cluster_ < 0.05 demonstrate that the observed between-group differences were partially attributable to increases in connectivity within the SMART group (Fig. 4), it was not sufficient to reveal which group drove the between-group differences within all of the identified regions shown in Fig. 3. Color maps for within- and between- group contrasts, uncorrected for multiple comparisons (*p*_voxel_ < 0.001; Figs. S1–S3) aided which group led to the observed group differences (i.e., Fig. 3). Surface maps for between-group contrast for connectivity changes according to the patterns of within-group changes (Fig. 5) clarified that the SMART group contributed predominantly to the observed group differences except in connectivity with the dLPFC and L IPS seeds. See Table S5 for more detailed information on temporal change patterns.

**Fig. 6:**
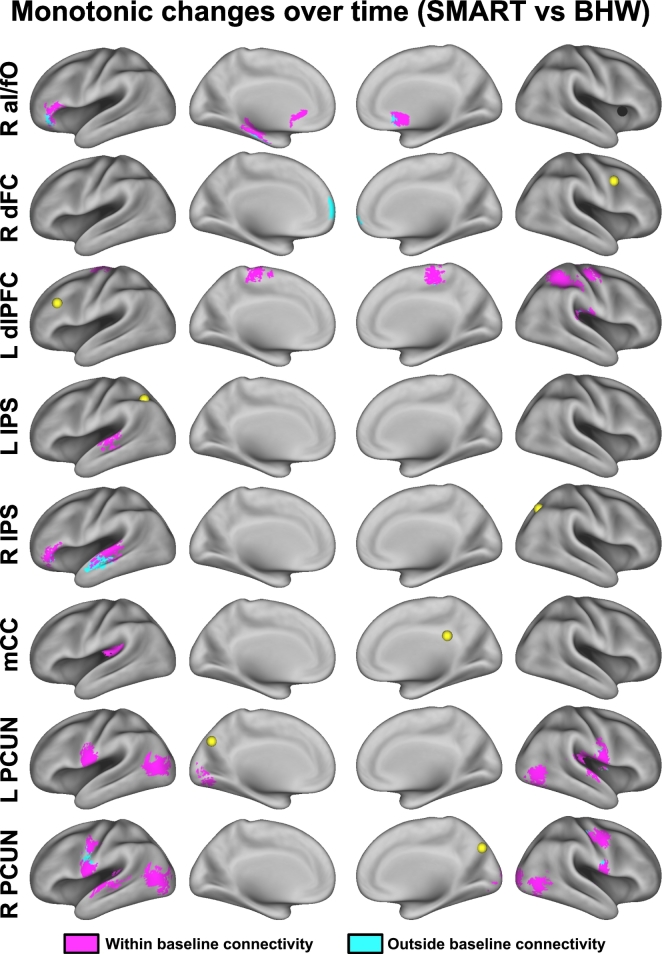
Colormaps for temporal changes in connectivity relative to baseline connectivity.

Color maps plotting the surface overlap of temporal changes in seed-based connectivity with the corresponding baseline seed-based connectivity revealed that SMART increased seed-based connectivity primarily within the regions where connectivity had already been established at baseline (Fig. 6). Though the brain regions where connectivity changes occurred within and outside baseline connectivity with the R aI/fO, R IPS, L PRECUN, and R PCUN seeds were spatially adjacent to each other, reductions in cognitive control networks connectivity in chronic TBI have been reported previously (Han et al., 2016). Similarly, though training-induced increases in connectivity with R dFC occurred outside baseline connectivity, in a previous study (Han et al., 2016) we identified reductions in connectivity between the cognitive control networks and default mode network in chronic TBI. Taken together, the brain regions in which connectivity changes occurred outside baseline connectivity *in chronic TBI* may fall within the connected regions present in *healthy* individuals.

#### 3.3.2 Aggregated results

**Fig. 7:**
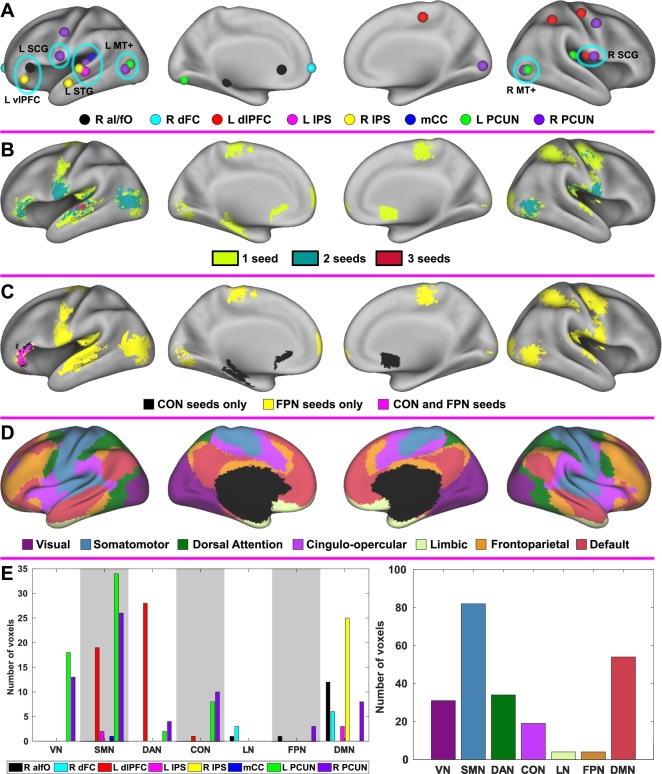
Composite maps across seeds. A: Foci maps. B: Connectivity overlap across seeds, C: Colormaps for temporal changes in connectivity according to the cognitive control networks. D: The Yeo atlas of large-scale resting-state networks (Yeo et al., 2011). E: The counts of voxels with statistically significant changes in connectivity according to the resting-state networks. SCG, subcentral gyrus; vlPFC, ventrolateral prefrontal cortex; STG, superior temporal gyurs; MT+, middle temporal complex; VN, visual network; SMN, somatomotor network; DAN, dorsal attention network; CON, cingulo-opercular network; LN, limbic network; FPN, fronto-parietal network; DMN, default mode network. See Fig. 1 for the other abbreviations.

Composite foci maps for the observed changes in connectivity following training revealed that training-induced increases in connectivity across multiple seed regions were prominent in the bilateral middle temporal complex and subcentral gyrus; left superior temporal gyrus and ventrolateral prefrontal cortex (Fig. 7A). Surface maps for connectivity overlap across seeds (Fig. 7B) further quantified that changes in connectivity overlapped up to 3 seeds. This illustrates the complex and unique patterns of training-induced changes in connectivity across seeds in individuals with TBI. The region where increases in connectivity occurred with both cognitive control networks (i.e., the left ventrolateral prefrontal cortex) corresponded to the default mode network (Fig. 7C–D). Increases in connectivity only with the cingulo-opercular network occurred in the subcortical regions (Fig. 7C). Similarly, the most of the regions that showed increases in connectivity only with the fronto-parietal network belonged to the visual, somatomotor, dorsal attention, and default networks (Fig. 7C–D). The voxel counts with statistically significant increases in connectivity according to large-scale resting-networks revealed that the distributions of these voxel counts differed across the seeds (Fig. 7E *left*). The aggregated counts of these voxels across the assessed seeds indicated that increases in cognitive control networks primarily occurred within the regions in the visual, somatomotor and default mode networks, not within the cognitive networks (Fig. 7E *right*).

### 3.4 Brain—behavior relationships

**Fig. 8:**
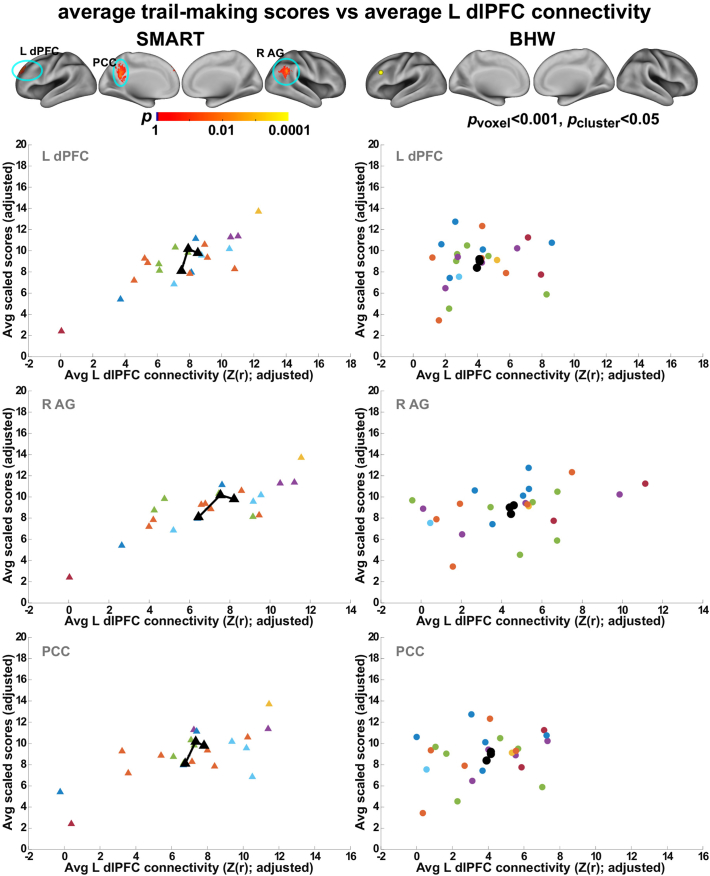
Associations between the average L dlPFC connectivity and average scores of the trail making test over time. Top row: Colormaps for statistically significant associations between the two measures. Other rows: Average L dlFPC connectivity versus average trail making scores within each of the clusters in the top row. Each colored triangle (circle) represents average L dlPFC and trail making score of each individual from the SMART (BHW) group, and black symbol represents group-averaged trajectory in the regions. dPFC, dorsal prefrontal cortex; AG, angular gyrus; PCC, posterior cingulate cortex. See Fig. 1, Fig. 2 for the other abbreviations.

**Fig. 9:**
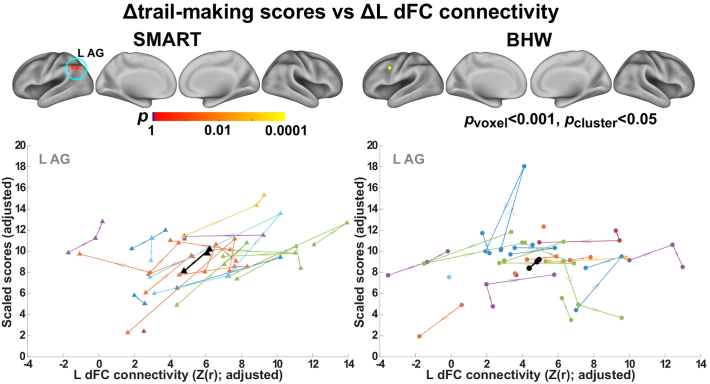
Associations between temporal changes in the trail making scores and changes in connectivity with L dFC. Top row: Colormaps for statistically significant associations between the two measures. Bottom row: Trajectories of each individuals (colored line) and group average (black line). See Fig. 1, Fig. 2, Fig. 8 for the other abbreviations.

We observed statistically significant (*p*_voxel_ < 0.001, *p*_cluster_ < 0.05) associations of trail making scores with fronto-parietal network connectivity within the SMART group (Fig. 8, Fig. 9). Positive association between the average L dlPFC connectivity over time and the average trail-making scores over time occurred within the regions of the default mode network (the posterior cingulate cortex; left dorsal prefrontal cortex; right angular gyrus) (Fig. 8). Positive associations indicated that the SMART participants with higher average trail making scores showed greater average L dlPFC connectivity with these regions. Temporal increases in trail-making scores were associated with increases in L dFC connectivity with the left angular gyrus within the default mode network (Fig. 9). See Table 4 for the detail information on these regions. Statistically significant associations between these two measures did not occur within the BHW group. Scatter plots for connectivity versus trail making test scores over time (Fig. 8, Fig. 9) confirmed the observed group analysis results.

**Table 4:** Regions showing statistically significant associations between connectivity and neuropsychological test scores within the SMART group (*p*_voxel_ < 0.001; *p*_cluster_ < 0.05).

#	Neuropsychological measure vs connectivity	Region	Major cluster	*p*_vox_	x	y	z
1	Average trail-making scores vs average L dlPFC connectivity	L Dorsal prefrontal cortex	1 (23 voxels)	<10^−5^	−18	42	38
2		R Angular gyrus	2 (22 voxels)	<10^−4^	58	−62	30
3		Posterior cingulate cortex	3 (18 voxels)	<10^−4^	−6	−50	26
4	*Δ*trail-making scores vs *Δ*L dFC connectivity	L Angular gyrus	4 (17 voxels)	<10^−4^	−50	−66	46

*Note*: See Fig. 1 for abbreviations and details.

### 3.5 Control analyses results

#### 3.5.1 Motion analysis results

The LME analysis on the average FD of each scan indicated that there were no systematic differences in subject motion between groups and across time within each of the groups (Table S6). Between-group differences in connectivity changes with FD covariates were consistent with main findings (Fig. S4), confirming that the uniform smoothing procedure controlled for the effects of head motion during rsfMRI scans.

#### 3.5.2 The effects of injury characteristics on connectivity

The LME analysis without covariates for residual psychiatric symptom severity yielded slightly weaker group contrasts (Fig. S5), compared to the group contrasts from the LME analysis with psychiatric symptom covariates (Fig. S1) at *p*_voxel_ < 0.001. However, the overall patterns were retained. The LME analysis with additional post-injury time covariate essentially replicated our findings on between-group contrasts for temporal changes in cingulo-opercular and fronto-parietal networks connectivity, indicating no significant effects of post-injury time on connectivity (Fig. S6).

#### 3.5.3 Assessment results for the subsets of the participants

The between-group contrast for changes in connectivity of the participants who underwent MRI scans at all time points (Fig. S7) was consistent with the main findings. This analysis confirmed that there was no systematic bias resulting from participant attrition. Neuropsychological assessment results for the participants who underwent MRI scans retained the patterns observed from the full sample, though it was underpowered due to the limited sample size of the subset (Table S7).

## 4 Discussion

We demonstrated changes in cingulo-opercular and fronto-parietal networks connectivity of individuals with chronic mild TBI following strategy-based cognitive training. We also identified the patterns of connectivity that were associated with neuropsychological performance over time. Our findings extend the rsfMRI literature on neuroplasticity in adults with clinical conditions. We also provided evidence of neuroplasticity in chronic TBI, isolating the patterns of brain responses that are associated with cognitive training. A strength of our study is the large sample size (N = 45; 109 rsfMRI scans) for connectivity analysis and comparisons with an active control group. Further, we assessed group-by-time interaction effects, frequently considered to be gold standard evidence for neural and behavioral changes following intervention (Thomas and Baker, 2013).

The current study provided evidence for brain responses to cognitive training for chronic TBI. Though several studies have reported the efficacy of cognitive training for TBI (Cicerone et al., 2006), there has been limited neuroscientific evidence for intervention-related effects on the brain. It is important to assess the effectiveness of TBI training from the cognitive neuroscience perspective (Cicerone et al., 2006; Kleim and Jones, 2008) given the pressing need for more research that may allow reliable measures of treatment efficacy and optimizing the effectiveness of cognitive training for TBI (Frieden et al., 2015). Brain regions comprising of the cingulo-opercular and fronto-parietal networks in this study were obtained from the patterns of sustained brain activity and start-cue-related activity during variety of cognitive control fMRI tasks in healthy individuals, respectively (Dosenbach et al., 2006). RsFC revealed that these brain regions were functionally connected. During cognitive control processes, the cingulo-opercular network is thought to be associated with the ability to maintain relevant goals, and the fronto-parietal network is thought to be associated with the ability to adjust goals (Dosenbach et al., 2007, Dosenbach et al., 2008). Several studies demonstrated that abnormality in the cingulo-opercular and fronto-parietal networks explains deficits to higher-order cognitive functions in various clinical populations (Bonnelle et al., 2012; Etkin et al., 2009; Jilka et al., 2014; Jolles et al., 2016; Sheffield et al., 2015; Silk et al., 2008; Trujillo et al., 2015; Wu et al., 2016). Taken together, our findings on increased cingulo-opercular and fronto-parietal networks connectivity after cognitive training for TBI (Fig. 3) highlight that previously impaired cognitive controls networks (i.e., cingulo-opercular network and fronto-parietal networks) by a TBI can be influenced by training-related neuroplasticity.

Our findings demonstrate the sensitivity and specificity of rsFC in assessing neuroplasticity following cognitive training for chronic TBI. The heterogeneity of TBI and limited sensitivity of conventional measures are major challenges to identify TBI-related abnormalities and the effects of cognitive training on the injured brain. As such, changes in neuropsychological performance following training may be difficult to capture due to the low sensitivity (Table 3). However, we observed statistically significant increases in rsFC following the SMART program (Fig. 3) and these changes occurred primarily within the SMART group (Figs. S2–3; Table S5) relative to the comparison BHW intervention. Further, within the SMART group, spatial patterns of changes in connectivity varied based upon seed locations (Fig. 3). Thus, our findings suggest that rsFC may serve as an effective neuroimaging-based biomarker of responses to training for TBI.

From the perspective of large-scale resting-state networks, connectivity changes after SMART primarily occurred at the level of between-network connectivity. Specifically, based on the Yeo atlas (Yeo et al., 2011), changes in the cingulo-opercular network primarily occurred between connectivity with the default mode network (Fig. 7). Similarly, changes in the fronto-parietal network connectivity primarily occurred in association with the visual, somatomotor, and default mode networks (Fig. 7). Interactions between brain networks are critical for successful cognitive control processes due to the diverse nature of control processes drawing from neural resources across the brain (Cocchi et al., 2013). Supporting this claim, a previous study reported interactions between the cingulo-opercular network and other brain regions during tasks involving other cognitive functions such as visuospatial attention and episodic memory (Sestieri et al., 2014). Further, network analyses of brain imaging data across 77 cognitive tasks from healthy individuals demonstrated that activity in brain regions of between-module connections increased when more cognitive components were engaged in a task. This indicates the importance of between-network connectivity for assessing “higher-order” cognitive functions (Bertolero et al., 2015). Individuals with TBI often show deficits in “higher-order” cognitive functions that require the integration of information across the brain (Sharp et al., 2014). Network analyses further revealed that TBI markedly disrupts between-network connectivity, yielding reduced efficiency of information processing (Han et al., 2014, Han et al., 2016). Taken together, increased between-network connectivity with the cingulo-opercular and fronto-parietal networks following SMART for TBI may indicate improved integration of information processing for higher-level cognitive functions.

The increases in cingulo-opercular and fronto-parietal networks connectivity after SMART align with previous literature on rsFC plasticity following training. A recent literature review suggests that training and practice strengthen functional connections between brain regions, as a majority of the previous studies reported increases in rsFC after training (Kelly and Castellanos, 2014). In the context of TBI, overall reductions in between-network connectivity after TBI have been reported (Han et al., 2014, Han et al., 2016) although the directionality of alterations in connectivity over the whole brain after a TBI is arguable. Thus, increases in between-network connectivity after training for TBI were not surprising. Positive associations between fronto-parietal network connectivity and card-sorting test performance over time within the SMART group (Fig. 8, Fig. 9) further supported the directionality of connectivity changes. Though the underlying biological mechanisms of increased cingulo-opercular and fronto-parietal networks connectivity after SMART for TBI remain unclear (potential mechanisms are discussed elsewhere (Tardif et al., 2016; Taubert et al., 2011)), our findings suggest that such increased rsFC with the cingulo-opercular and fronto-parietal networks may indicate improved neural network efficiency supporting cognitive control.

Regarding brain-behavior relationships, associations between the trail-making test scores and cognitive control networks (Fig. 8, Fig. 9) occurred within the regions of the default mode network (i.e., angular gyrus and posterior cingulate cortex; the left dorsal prefrontal cortex). Trail-making involves a combination of working memory, task-switching and visuoperceptual abilities (Sánchez-Cubillo et al., 2009), indicating that cognitive control is an essential construct involved in successfully coordinating abilities to achieve better performance. Thus, it was not surprising to observe correlations between the trail-making scores and fronto-parietal network connectivity within the SMART group. The majority of these correlations occurred in the regions where the fronto-parietal and default mode networks interact. Though an antagonistic relationship between the fronto-parietal and default networks has frequently been described, recent reports have challenged this view. Recent studies have demonstrated that interactions between the fronto-parietal and default mode network activity support goal-directed cognition (Spreng et al., 2010, Spreng et al., 2014) and individuals with greater cooperation between the fronto-parietal and default mode networks showed faster reaction times during a goal-directed recollection task (Fornito et al., 2012). Further, positive correlations between the default mode network connectivity and trail-making scores in patients with mild TBI have been reported (Zhou et al., 2012). A few studies have reported brain activity within the regions of the fronto-parietal network during fMRI versions of the trail-making test (Moll et al., 2002; Zakzanis et al., 2005). Taken together, the observed brain-behavior relationship was relevant to findings in previous studies.

The present study has some limitations. First, we did not directly compare the cognitive control network connectivity of the participants with a healthy control group. As such, we were not able to establish how close the patterns of cognitive networks were to those of healthy individuals after training. However, we confirmed that the TBI participants who initially performed poorly on neuropsychological performance reached the level of healthy individuals after SMART based on scaled scores (Fig. 2C–D). Thus, the study does demonstrate the efficacy of cognitive training for TBI despite of this limitation. Second, the attrition rate was relatively high (total number of available rsfMRI scans was 109 instead of 135). This is a typical limitation of on-site cognitive training in this population, as participants required a substantial commitment to attend multiple training sessions for 8 weeks. We performed additional analyses to confirm that participant attrition did not bias our main findings (Fig. S7). Nonetheless, the findings would be more convincing if we had achieved a lower attrition rate. Third, the effects of SMART on connectivity among large-scale networks other than cognitive control networks are still unknown. Though the cingulo-opercular and fronto-parietal networks would be the most relevant network to assessed training program, SMART is an integrative training that aims to multiple domains of cognitive functions such as abstract reasoning, selective attention, and cognitive control (Vas et al., 2011). We employed the integrated training program for this study because the best evidence for improvements in broad health-related outcomes are provided by studies in comprehensive cognitive training (Cicerone et al., 2011). Thus, it is possible that the SMART could induce changes between other resting networks in these individuals. In this future, systematic assessment of several resting-state networks over the whole brain would be informative in this regard. Lastly, the interpretation of the current finding regarding increased connectivity of cognitive control networks with other large-scale resting-state networks is less straightforward than changes in connectivity within cognitive control networks. However, cognitive control networks are connected with multiple large-scale resting-networks (Dosenbach et al., 2006, Dosenbach et al., 2007) due to their role in coordinating multiple cognitive processing (i.e., cognitive control). Based on resting-state functional connectivity alone, it is difficult to precisely interpret changes in connectivity between cognitive control networks and other networks. Although, the interpretation of the observed changes in connectivity of cognitive control networks with other large-scale resting-state networks is less straightforward, the findings are relevant in the context of TBI. As discussed before, TBI markedly disrupts between-network connectivity compared to within-network connectivity (Han et al., 2014, Han et al., 2016) and between-network connectivity plays a critical role in ‘higher-order’ cognitive functions (Bertolero et al., 2015). The SMART program induced changes in connectivity between cognitive control networks and other large-scale resting-state networks, which was related to the disruption patterns of connectivity in TBI. Nonetheless, a future study in task-state functional connectivity of our participants would allow us to better understand the implications of current findings in training-induced functional connectivity.

In conclusion, we utilized resting-state functional connectivity to elucidate neuroplasticity following cognitive training for chronic TBI. Specifically, we demonstrated that strategy-based cognitive training led to increases in connectivity with the cingulo-opercular and fronto-parietal networks in individuals with chronic TBI relative to an information-based training group, even 3 months after training was completed. Our findings suggest that training-induced neuroplasticity continues through the chronic phases of TBI, and resting-state functional connectivity may be a potential neuroimaging biomarker for evaluating cognitive training for chronic TBI linked to improved cognitive control.

## Appendix A Supplementary data

Supplementary material Image 1
