# Supplementary material for: Neuroplasticity of cognitive control networks following cognitive training for chronic traumatic brain injury
Source: Neuroimage Clin. 2018 Jan 31;18:262–78. doi: 10.1016/j.nicl.2018.01.030 (PMC5987796; doi:10.1016/j.nicl.2018.01.030)
Supplement: Supplementary file 1 — Supplementary material [file mmc1.pdf]

Supplementary Material

Title: Neuroplasticity of cognitive control networks following cognitive training for chronic traumatic brain injury[†]

Authors: Kihwan Han[1,*], David Martinez[1], Sandra B Chapman[1], Daniel C Krawczyk[1,2]

Affiliations:

[a]Center for BrainHealth®, School of Behavioral and Brain Sciences, The University of Texas at Dallas

[b]Department of Psychiatry, University of Texas Southwestern Medical Center

Corresponding author:

Kihwan Han, PhD

Center for BrainHealth®, School of Behavioral and Brain Sciences

University of Texas at Dallas

2200 West Mockingbird Lane, Mail Stop: CBH

Dallas, TX 75235, USA

Email: kihwan.han@utdallas.edu, Tel: 1-972-883-3429, Fax: 1-214-905-3026

Co-authors:

Sandra B Chapman, PhD (Email: schapman@utdallas.edu)
Daniel C Krawczyk, PhD (Email: daniel.krawczyk@utdallas.edu)

[†]The views and opinions expressed in this article are those of the authors and do not reflect the official policy or position of the Department of the Army, Department of the Air Force, Department of Defense or United States Government.

Supplementary Tables

Table S1. Changes in residual psychiatric symptom severity

Psychiatric measure	SMART (N=26)				BHW (N=30)				SMART vs BHW
	TP_1	TP_2	TP_3	*p*-values (M, NM)[a]	TP_1	TP_2	TP_3	*p*-values (M, NM)[a]	*p*-values (M, NM)[a]
BDI-II	23.3±9.3	15.3±10.2	13.2±9.2	<0.01*, 0.05*	18.5±12.6	13.8±11.4	12.9±10.9	<0.01*, >0.1	>0.1 , >0.1
PCL-S	46.4±16.5	43.5±17.8	37.8±16.6	<0.01*, >0.1	46.5±19.0	39.6±19.5	39.0±20.4	0.01*, >0.1	>0.1 , >0.1

Note: SS, Scaled scores; M, Monotonic; MN, Non-monotonic. See Tables 1, 2 for other abbreviations.

[a] * represents $p < 0.05$.

Table S2. Within-group changes in neuropsychological test scores

Neuropsychological measures	*p*-values (M, NM)[a]	
	SMART (N=26)	BHW (N=30)
Card-sorting (SS)	<0.01*, 0.02*	0.01*, <0.01*
Trail-making (SS)	0.03*, 0.01*	>0.1 , >0.1

Note: See Table 3 for abbreviations.

[a] * represents $p < 0.05$.

Table S3. The number of civilians and veterans per time point by group

	Neuropsychological assessments		Resting-state fMRI scans	
Time point	SMART[a]	BHW[a]	SMART[a]	BHW[a]
TP_1	17, 9	18, 12	14, 6	14, 7
TP_2	16, 9	16, 9	14, 6	14, 5
TP_3	12, 7	15, 9	9, 5	10, 5

[a] Civilians, Veterans

Table S4. The distribution of primary cause of injury per time point by group

	Neuropsychological assessments		Resting-state fMRI scans	
Time point	SMART[a]	BHW[a]	SMART[a]	BHW[a]
TP_1	5, 2, 2, 8, 7, 2	5, 6, 5, 5, 7, 2	2, 1, 2, 7, 6, 2	4, 5, 4, 4, 2, 2
TP_2	5, 2, 2, 7, 7, 2	4, 6, 4, 4, 6, 1	3, 1, 2, 6, 6, 2	3, 5, 3, 5, 2, 1
TP_3	4, 2, 1, 5, 6, 1	3, 5, 4, 5, 6, 1	2, 1, 1, 5, 5, 0	4, 3, 3, 2, 3, 0

[a] blast, blunt force trauma, fall, athletic impacts, vehicle accidents, combined

Table S5. Regions showing statistically significant group differences in changes in cognitive control networks connectivity (SMART > BHW; $p_{\text{voxel}} < 0.001$; $p_{\text{cluster}} < 0.05$).

#	Seed	Region	Major cluster	Z[a]	x[b]	y[b]	z[b]	Temporal change pattern[c]	
								SMART	BHW
1	R aI/fO	L Parahippocampal gyryus	1 (21 voxels)	4.5	-22	-26	-10	–	↓[d]
2		L Ventrolatral prefrontal cortex	2 (19 voxels)	4.0	-52	27	-8	↑[d]	–
3		Ventral anterior cingulate cortex	3 (16 voxels)	4.6	-2	22	-2	↑[d]	–
4	R dFC	Anterior prefrontal cortex	4 (15 voxels)	3.9	-6	66	-10	↑	–
5	L dlPFC	R Intraparietal sulcus	5 (57 voxels)	4.8	38	-46	50	–	↓
6		Paracentral lobule	6 (48 voxels)	4.1	6	-22	58	↑[d]	–
7		R Precentral gyrus	7 (19 voxels)	4.9	34	-14	58	–	↓[d]
8		R Subcentral gyrus	8 (15 voxels)	4.1	54	-14	18	–	↓
9	L IPS	L Superior temporal gyrus	9 (12 voxels)	4.4	-58	-26	2	–	–
10	R IPS	L Superior temporal gyrus	10 (22 voxels)	4.3	-70	-22	-6	↑[d]	–
11		L Ventrolateral prefrontal cortex	11 (14 voxels)	3.9	-46	34	-14	↑[d]	–
12		L Superior temporal gyrus	12 (12 voxels)	4.2	-54	-10	-14	–	–
13	mCC	L Posterior insula	13 (18 voxels)	4.2	-46	-42	14	↑[d]	↓[d]
14	L PCUN	R Subcentral gyrus	14 (44 voxels)	4.5	62	-6	10	↑[d]	–
15		L Subcentral gyrus	15 (30 voxels)	4.7	-62	-6	14	↑[d]	↓[d]
16		L Middle temporal complex	16 (27 voxels)	3.8	-42	-74	6	↑[d]	–
17		R Middle temporal complex	17 (23 voxels)	3.9	42	-70	-6	↑[d]	–
18		R Superior temporal gyrus	18 (14 voxels)	4.0	38	-30	18	↑[d]	–
19		L Lingual gyrus	19 (13 voxels)	3.7	-14	-78	-2	↑	–
20	R PCUN	R Cuneus	20 (37 voxels)	4.0	22	-86	2	↑[d]	–
21		L Middle temporal complex	21 (36 voxels)	4.2	-42	-62	6	↑	–
22		L Superior temporal gyrus	22 (28 voxels)	4.5	-50	-30	2	↑[d]	↓[d]
23		L Subcentral gyrys	23 (26 voxels)	4.4	-62	-6	14	↑	–
24		R Precentral gyrus	24 (20 voxels)	4.4	50	-2	50	↑[d]	–
25		R Middle temporal complex	25 (19 voxels)	4.2	38	-74	-6	↑	–
26		R Subcentral gyrus	26 (13 voxels)	4.2	62	-6	10	↑[d]	–
27		L Precentral gyrus	27 (13 voxels)	4.2	-54	-6	46	↑[d]	–

Note: L, Left; R, Right; aI/fO; anterior insula/frontal operculum; dFC, dorsal frontal cortex; dlPFC, dorsolateral prefrontal cortex; IPS, inferior parietal sulcus; mCC, middle cingulate cortex; PCUN, precuneus..

[a] Local peak whose distance from other peaks within the cluster was longer than 30 mm.

[b] The Montreal Neurological Institute (MNI) space.

[c] '↑', '↓' and '–' symbols indicate monotonic increases, monotonic decreases and no significant changes, respectively.

[d] Within-group changes at $p_{\text{voxel}} < 0.001$.

Table S6. The amount of subject motion during fMRI scans per time point by group

Measure	SMART (N=21)			BHW (N=24)			*p* <0.05
	TP_1	TP_2	TP_3	TP_1	TP_2	TP_3	
FD (mm)	0.20 ± 0.08	0.21 ± 0.07	0.19 ± 0.10	0.21 ± 0.08	0.23 ± 0.10	0.23 ± 0.09	none

Note: FD, Framewise displacement.

Table S7. Neuropsychological assessment results for the participants who underwent MRI scans

Neuropsychological measure	SMART (N=21)			BHW (N=24)			p-values
	TP_1	TP_2	TP_3	TP_1	TP_2	TP_3	(M, NM)[a]
Card sorting (SS)	9.7±2.6	11.7±2.1	11.9±2.2	9.7±2.9	11.4±3.0	11.2±3.4	>0.1 , >0.1
Trail making (SS)	7.9±2.8	9.8±3.0	9.8±1.9	8.8±2.6	9.2±2.9	8.5±2.6	0.07[†], >0.1

See Tables 3 for abbreviations.

[a] † represents $p < 0.1$.

Supplementary figures

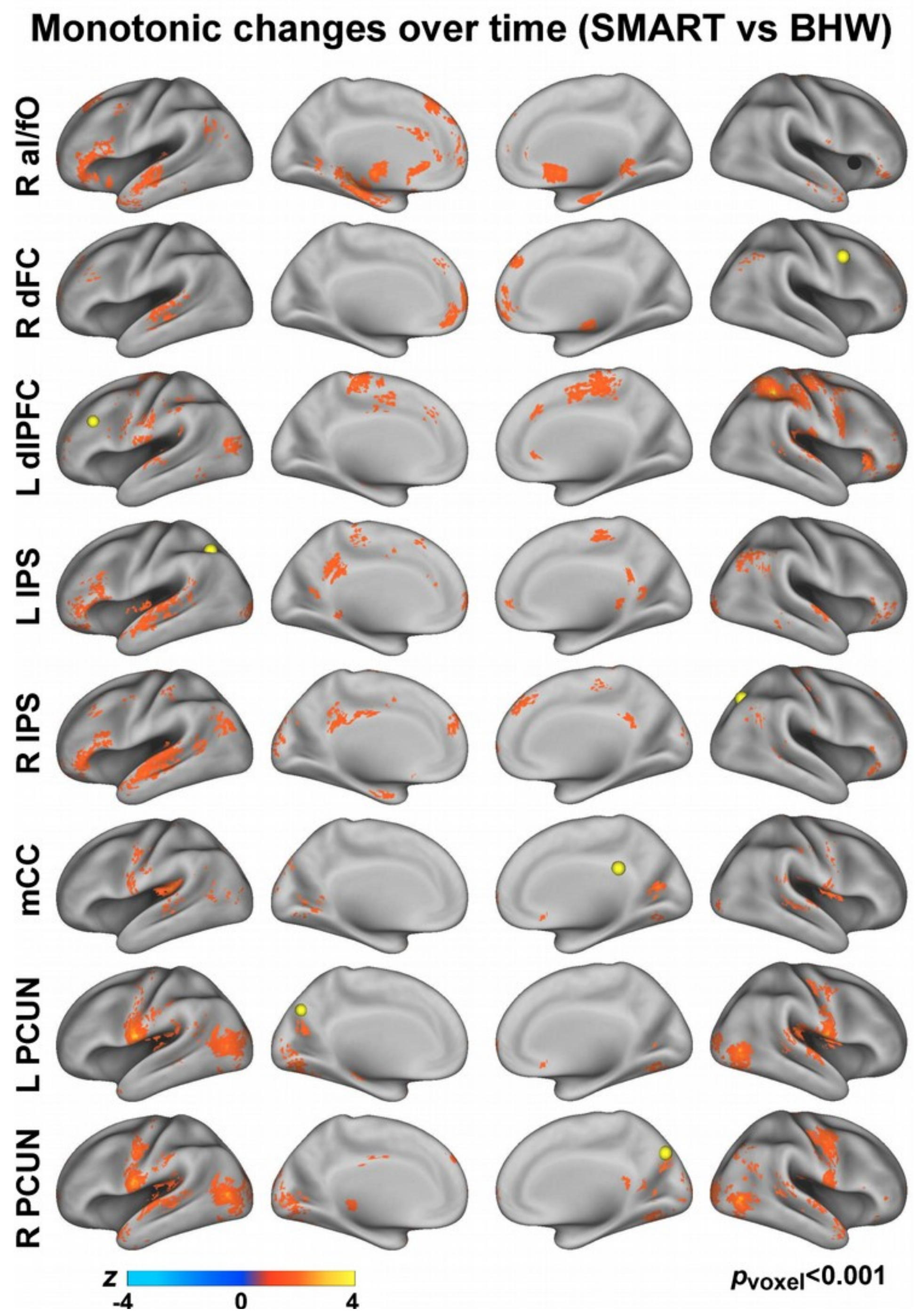

Figure S1. Between-group contrast maps for changes in connectivity over time (uncorrected).

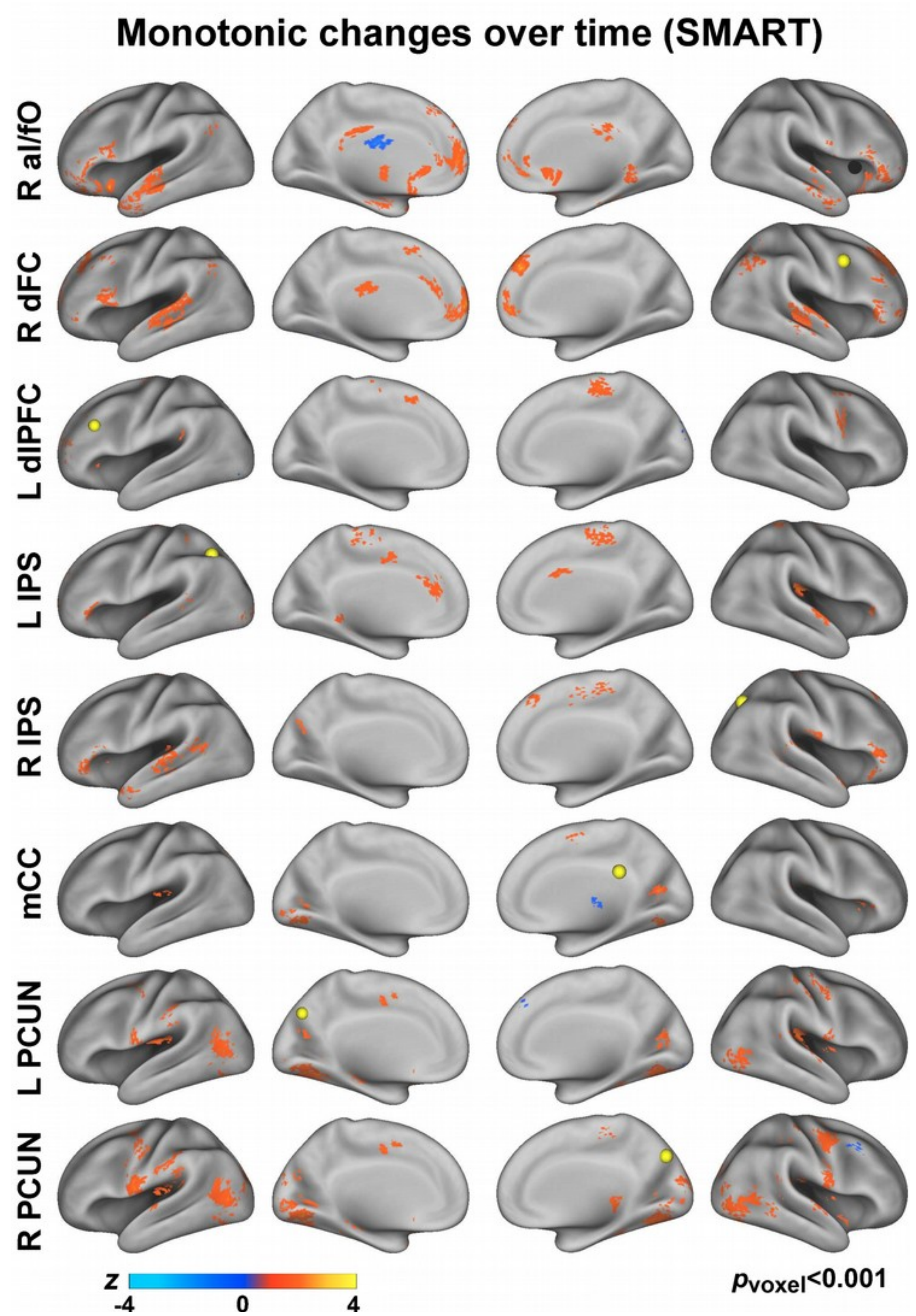

Figure S2. Within-group contrast maps for changes in connectivity over time for the SMART group (uncorrected).

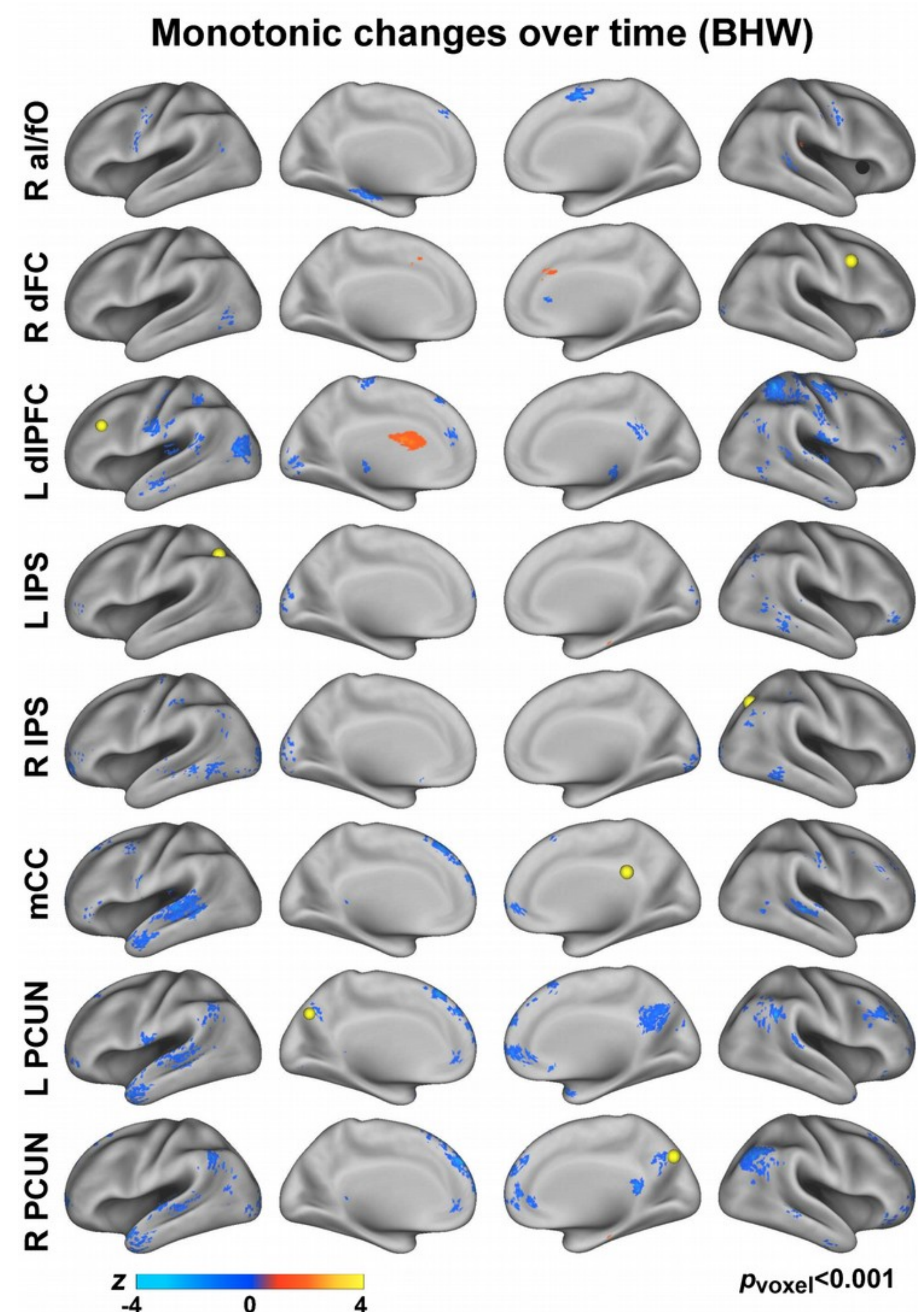

Figure S3. Within-group contrast maps for changes in connectivity over time for the BHW group (uncorrected).

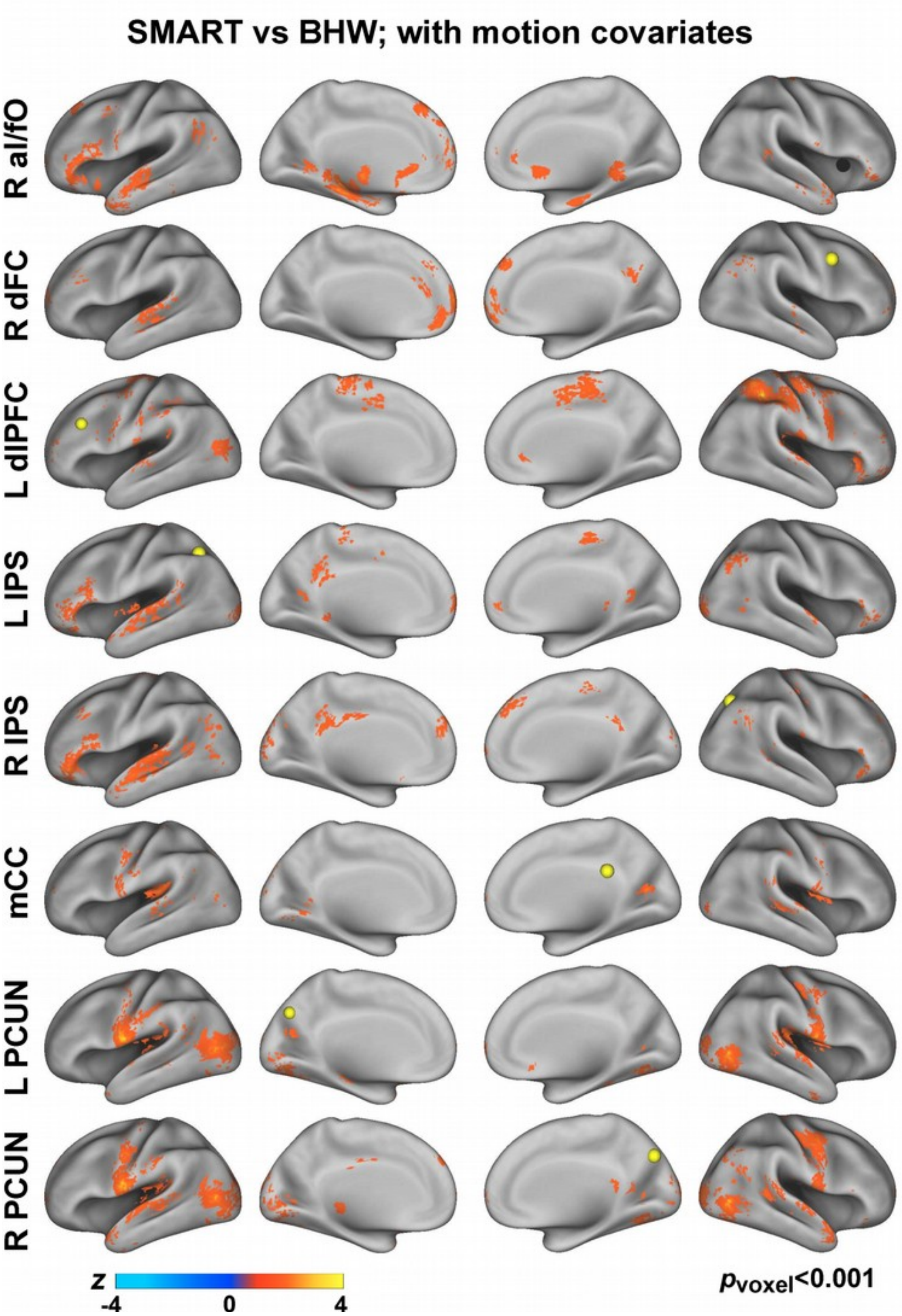

Figure S4. Between-group contrast maps for changes in connectivity over time with covariates for framewise displacement (uncorrected).

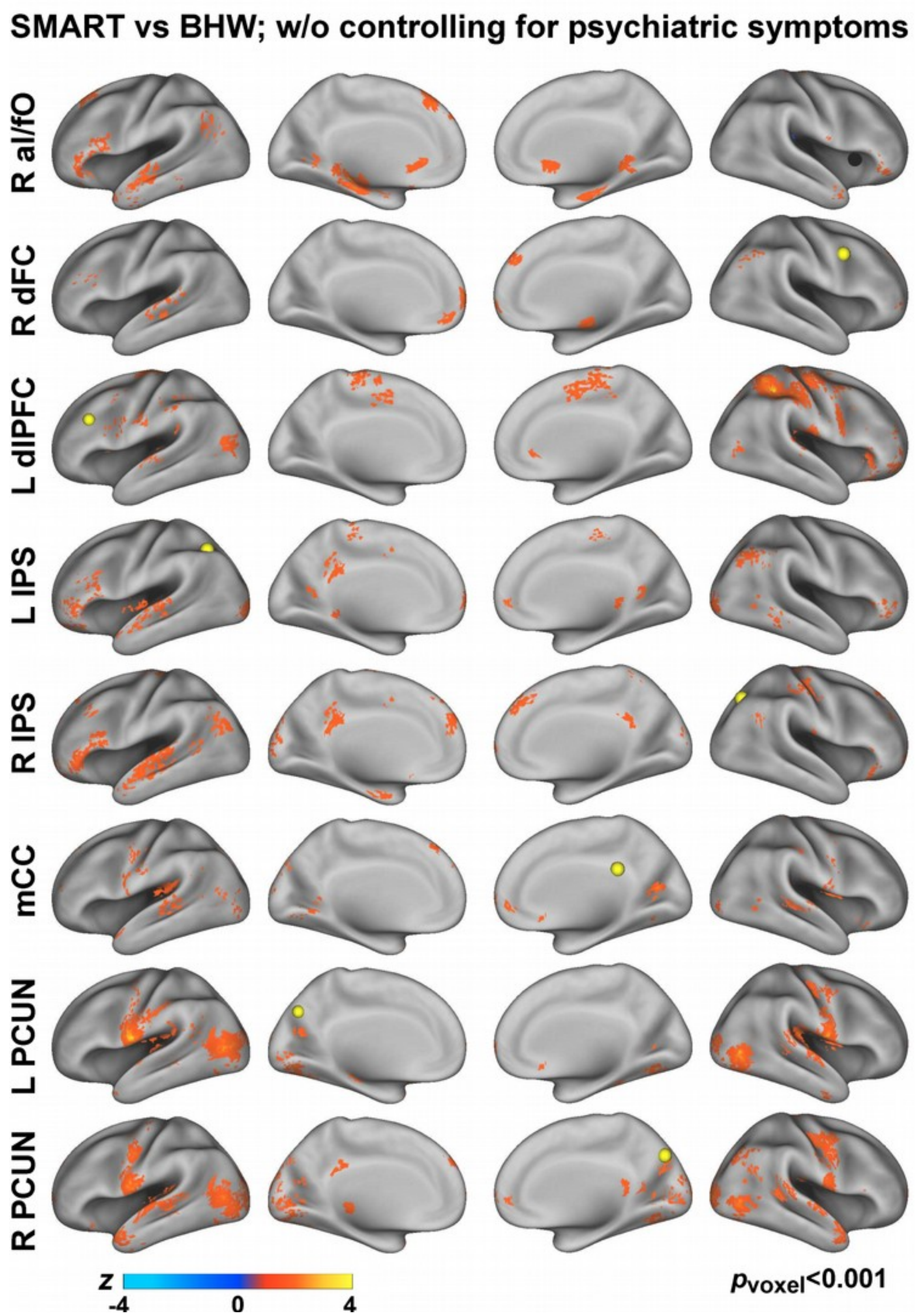

Figure S5. Between-group contrast maps for changes in connectivity over time without covariates for residual psychiatric symptom severity (uncorrected).

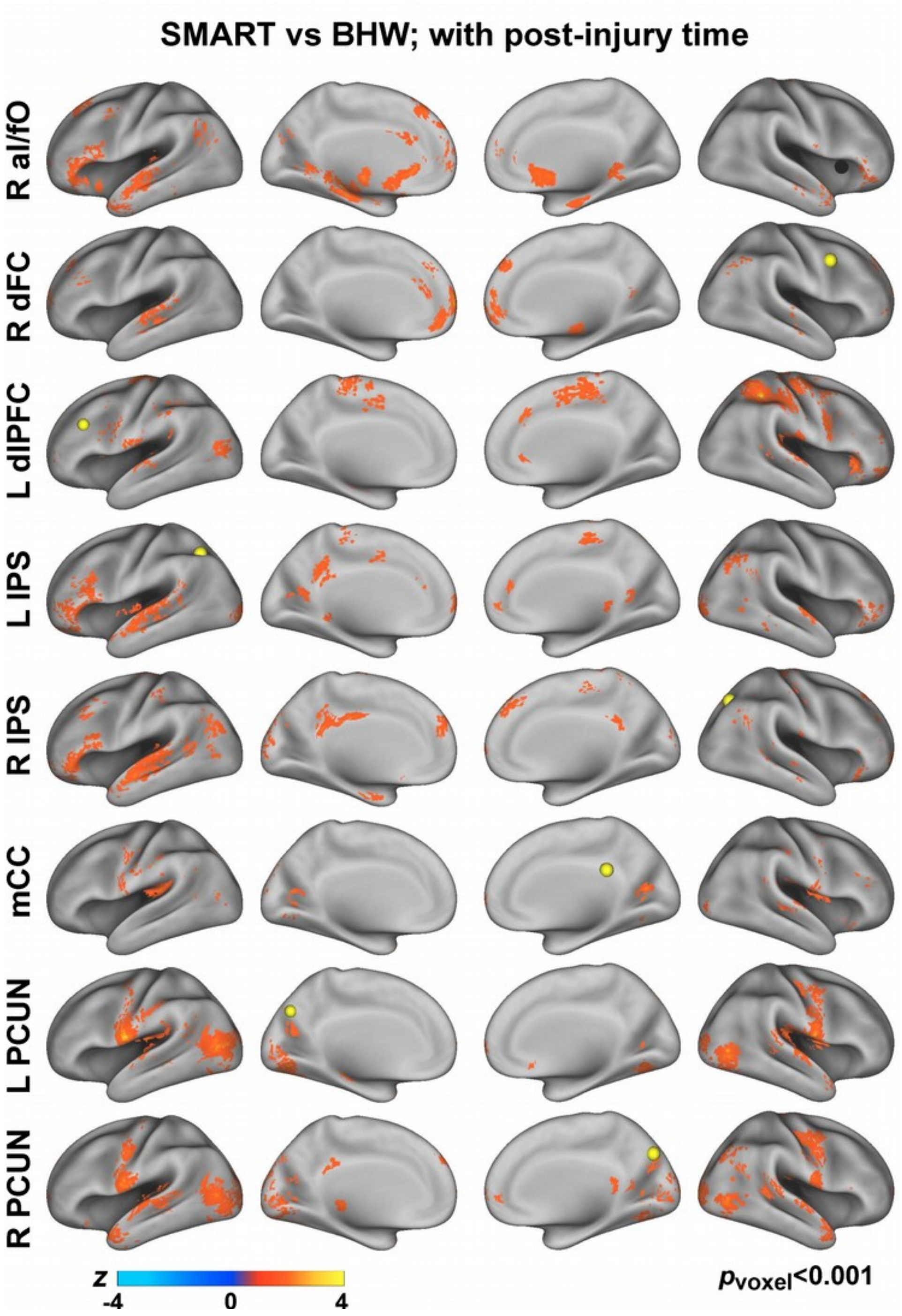

Figure S6. Between-group contrast maps for changes in connectivity over time with post-injury time covariates (uncorrected).

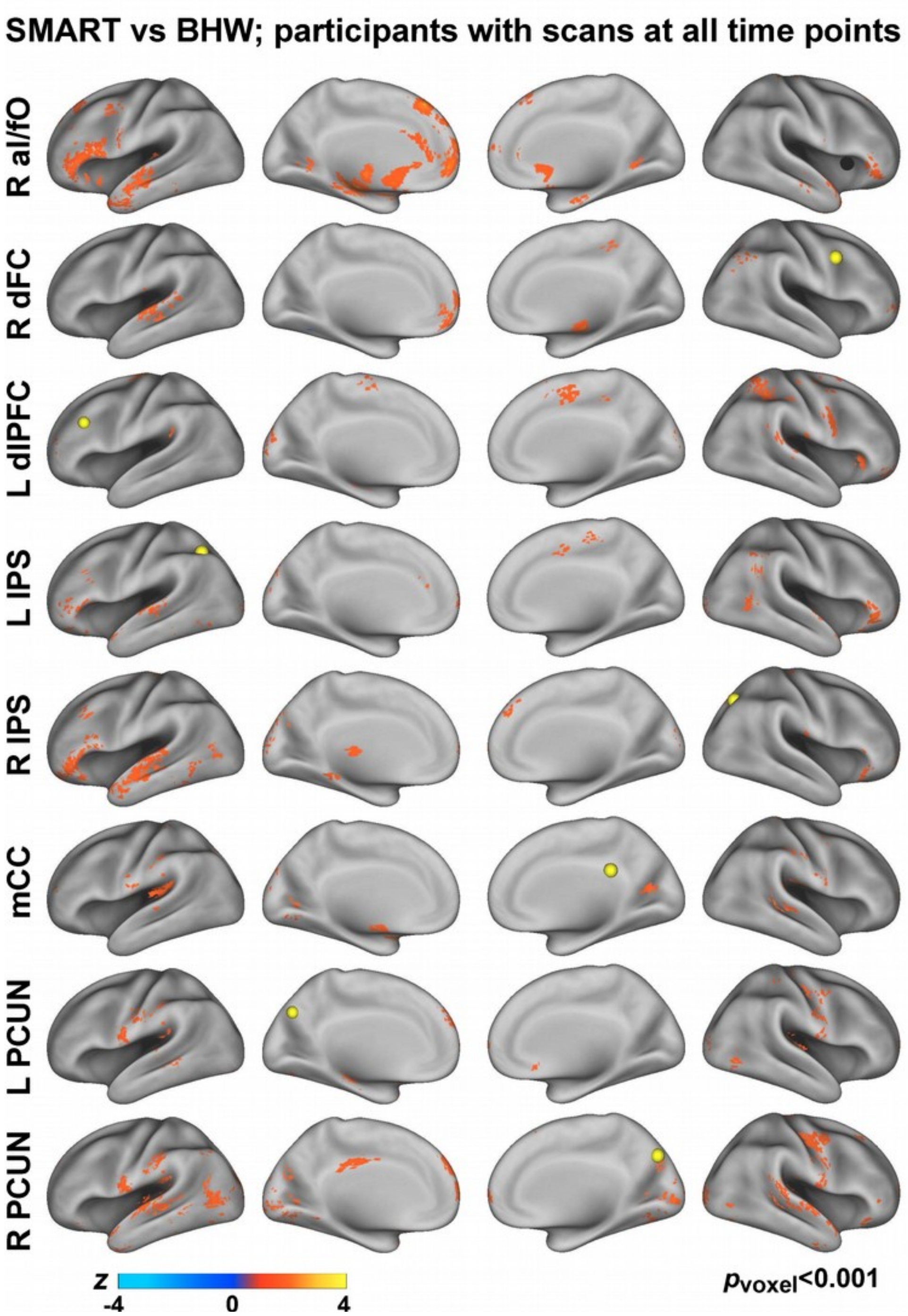

Figure S7. Between-group contrast maps for changes in connectivity of the subset of the participants who underwent MRI scans at all time points (uncorrected).
